# 

**Published:** 1890-04

**Authors:** 


THE MEDICAL ASSOCIATION OF THE STATE OF ALABAMA will hold its next meeting in Birmingham, April 8, 9, 10, and 11, 1890. Among the several titles of value that are announced, we note that Dr. W. E. B. Davis, of Birmingham, will read a paper entitled, A Study of the Treatment of Local and General Peritonitis.

---

THE NATIONAL ASSOCIATION OF RAILWAY SURGEONS will hold its second annual meeting at Kansas City, Mo., commencing Thursday, May 1, 1890.

---

THE TENNESSEE STATE MEDICAL SOCIETY will hold its annual meeting in Memphis, April 8, 9, and 10, 1890.

---

THE IOWA STATE MEDICAL SOCIETY will hold its thirty-ninth annual meeting at Des Moines, April 16, 17, and 18, 1890.

---

### Personal.

### BOOKS AND PAMPHLETS RECEIVED.

Practical Electricity in Medicine and Surgery. By G. A. Liebig, Jr., Ph. D., Assistant in Electricity, Johns Hopkins University, etc., and George H. Rohé, M. D., Prof. of Obstetrics and Hygiene, College of Physicians and Surgeons, Baltimore, etc. Profusely illustrated. 8vo. pp. viii., 383. Philadelphia and London: F. A. Davis, publisher. 1890. Price, $2.00.

The Neuroses of the Genito-Urinary System in the Male, with Sterility and Impotence. By Dr. R. Ultzman, Professor of Genito-Urinary Diseases in the University of Vienna. Translated by Gardner W. Allen, M. D., Surgeon to the Genito-Urinary Department, Boston Dispensary. 12mo pp. 160. Philadelphia and London: F. A. Davis. 1889. Price, $1.00.

A Text-Book on Diseases of the Eye. By Henry D. Noyes, A. M., M. D., New York: William Wood & Company. 1890. Imperial octavo. Price, $6.00, Muslin; $7.00, Half Russia.

Essentials of Disease of the Skin, including the Sypholodermata. Arranged in the form of questions and answers, prepared especially for students of medicine. By Henry W. Stelwągaon, M. D., Ph. D., Attending Physician to the Philadelphia Dispensary for Skin Diseases, etc. With seventy-four illustrations. Philadelphia: W. B. Saunders. 1890.

The Examination of Urine, Chemical and Microscopical, for Clinical Purposes. By Lawrence Wolff, M. D., Physician to the German Hospital of Philadelphia, etc. Colored plate and numerous illustrations. Saunders' Question Compends. Philadelphia: W. B. Saunders. 1890. Price, 75 cents.

Essentials of Gynecology, arranged in the form of questions and answers, prepared especially for students of medicine. By Edwin B. Cragin, M. D., Attending Gynecologist to the Out-Patient Department of Roosevelt Hospital, etc. With fifty-eight illustrations. Philadelphia: W. B. Saunders. 1890.

What are the Rational Limitations of Intra-Uterine Therapeutics? By Andrew F. Currier, M. D. Reprint from the New York *Medical Journal* for February 15, 1890.

Thirteenth Annual Report of the Medical Superintendent of the State Asylum for Insane Criminals, Auburn, N. Y., for the year ending September 30, 1889. Auburn: Wm. J. Moses, Printer.

Proceedings and Addresses at a Sanitary Convention held at Pontiac, Mich., Oct. 17 and 18, 1889. Proceedings and Addresses at a Sanitary Convention held at Vicksburg, Mich., December 5 and 6, 1889. (Supplements to the Report of the Michigan State Board of Health for 1889.) By authority. Lansing: Davens D. Thorp. 1890.

The Animal Suture: Its Place in Surgery. By Henry O. Marcy, A. M., M. D., L. L. D. Reprint from the Transactions of the American Association of Obstetricians and Gynecologists, Philadelphia. Wm. J. Dornan. 1889.

The Cure of Hemorrhoids by Excision and Closure with the Buried Animal Suture. By Henry O. Marcy, A.M., M. D., L. L. D., Boston, Mass. Reprinted from the Annals of Surgery, 1889.

---

### Literary and Other Notes.

THE *Cosmopolitan* for April presents an unusually interesting number. An article on The Fighting Forces of Germany, by Pultney Bigelow, that is most handsomely and accurately illustrated, opens a series of most entertaining and instructive magazine articles. The Land of the White Elephant, by Frank G. Carpenter, is another attractively written and beautifully illustrated paper. But what shall be said of Elizabeth Bisland's A Flying Trip Around the World? The chaste and elegantly simple English with which Miss Bisland tells her story, the first stage of which is here related, at once charms the reader and wins a sympathy for a young woman of culture and refinement, who was persuaded, much against her will, to make a tour that has suddenly brought her out into the blazing sunlight of publicity. How well she stands the glare, and with what womanly modesty she endures a distasteful notoriety for the sake of the cause in which she is embarked, can only be ascertained by reading her own story as told in the magazine to which she is so devoted.

The other attractions of the number we must perforce omit to mention for lack of space.

THE production of Ernest Reyer's new opera, "Salammbo," at Brussels, is the most important musical event that has thus far happened this year in Europe. A comprehensive account of this remarkable work, together with the estimates placed upon it by the best European critics, a bright personal sketch of the composer, an admirable portrait of him, and a reproduction of the music of one of the gems of the score constitute the leading attraction of the *Transatlantic* of March 15th. Almost equally remarkable is a review in the same issue of the Socialist party in Germany, which the recent elections in that country brought forward so prominently. The conclusion of Guy de Maupassant's "Vagrant Life," the continuation of the serial, "On the Mountain," a new criticism of Zola by the great Russian reviewer, Michailovsky, and an account of the discovery of a new Rembrandt in France, complete an attractive table of contents. 328 Washington street, Boston. $2.00 a year.

---

GYNECOLOGY.—There is probably no word in such common use that is so abused in its pronunciation as gynecology, and we are pleased to observe such a simple rule for its pronunciation as Dr. Hartshorn gives in his review of Billings' Dictionary in the April number of the *American Journal of Medical Sciences*. Says Dr. H.:

> It is to be wished that Dr. Billings, like Dr. Joseph Thomas in his excellent *Medical Dictionary*, had indicated the sound of g at the beginning of words. He might thus have aided in the now frequent mispronunciation of gynecology, which ought to have in its first syllable the sound of gy, as in gymnastics; not, as we often hear it, like the sound of gui in guide.

---

P. BLAKISTON, SON & CO., Philadelphia, published, about March 15th, a new Medical Dictionary, by George M. Gould, A.B., M.D. It is a compact one-volume book, containing several thousand new words and definitions, collected from recent medical literature, while the total number of words is beyond that in any similar book. It includes also elaborate and useful tables of the Bacilli, Leucomaïnes, Ptomaïnes, Micrococci, etc.; of the Arteries, Nerves, etc., and of the Mineral Springs of the United States, together with other collateral information.

---

TRANSACTIONS OF SPECIAL SOCIETIES FOR 1889.—A number of these volumes have already been issued, and are offered for sale through the book trade, or will be furnished on application to the printer, Mr. William J. Dornan, 100 North Seventh street, Philadelphia. Mr. Dornan is probably the most experienced medical society transactions printer in the country, and we invite attention to his advertisement, on page xxx.

